# Role of leucine-rich repeat kinase 2 in severe acute pancreatitis

**DOI:** 10.3389/fimmu.2024.1364839

**Published:** 2024-02-19

**Authors:** Yasuo Otsuka, Kosuke Minaga, Masatoshi Kudo, Tomohiro Watanabe

**Affiliations:** Department of Gastroenterology and Hepatology, Kindai University Faculty of Medicine, Osaka-Sayama, Osaka, Japan

**Keywords:** leucine-rich repeat kinase 2 (LRRK2), pancreatitis, fungi, tumor necrosis factor alpha (TNF-α), interleukin 6 (IL-6)

## Abstract

**Introduction:**

Intrapancreatic activation of trypsinogen caused by alcohol or high-fat intake and the subsequent autodigestion of the pancreas tissues by trypsin are indispensable events in the development of acute pancreatitis. In addition to this trypsin-centered paradigm, recent studies provide evidence that innate immune responses triggered by translocation of intestinal bacteria to the pancreas due to intestinal barrier dysfunction underlie the immunopathogenesis of acute pancreatitis. Although severe acute pancreatitis is often associated with pancreatic colonization by fungi, the molecular mechanisms linking fungus-induced immune responses to the development of severe acute pancreatitis are poorly understood. Leucine-rich repeat kinase 2 (LRRK2) is a multifunctional protein that mediates innate immune responses to fungi and bacteria. Mutations in *Lrrk2* is a risk factor for Parkinson’s disease and Crohn’s disease, both of which are driven by innate immune responses to gut organisms.

**Discussion:**

In this Minireview article, we discuss how activation of LRRK2 by the recognition of fungi induces severe acute pancreatitis.

## 1 Introduction

Acute pancreatitis, which is usually triggered by excessive alcohol intake, a high-fat diet, or gallstones, is a leading cause of urgent hospital admission (1–3). Although most patients with acute pancreatitis have a self-limiting clinical course, approximately 20% of patients develop a life-threatening form of acute pancreatitis, called severe acute pancreatitis (SAP), which is characterized by pancreatic necrosis and multiple organ failure. Given the high incidence of mortality (20%) in patients with SAP (1–3), curative treatment for SAP and/or a method of preventing progression of acute pancreatis to SAP is an unmet medical need.

Colonization of the pancreas by gut bacteria and/or fungi as a result of increased intestinal permeability plays a critical role in the development of pancreatic necrosis and multiple organ failure in patients with SAP (1–3). Consistent with this notion, we and others have shown that experimental acute pancreatitis requires proinflammatory cytokine responses induced by the sensing of bacterial components by pattern recognition receptors (PRRs) expressed in pancreatic acinar cells, macrophages, or dendritic cells (DCs) (4–6). Mice deficient in nucleotide-binding oligomerization domain 1 (NOD1) or Toll-like receptor 4 (TLR4), both of which are representative PRRs that recognize cell wall components derived from gram-negative gut bacteria, are resistant to experimental acute and chronic pancreatitis (4–6). Recent studies have elucidated some molecular mechanisms by which innate immune responses to gut bacteria cause acute pancreatitis. However, understating of fungus-induced immune responses underlying the immunopathogenesis of acute pancreatitis is limited. Leucine-rich repeat kinase 2 (LRRK2) is a multifunctional protein with the ability to induce autophagy and proinflammatory cytokine responses on sensing zymosan, a fungal cell wall component (7, 8). Polymorphisms in *Lrrk2* are associated with Parkinson’s disease (PD) and Crohn’s disease (CD), both of which are triggered by an excessive innate immune response to intestinal microorganisms (7, 8). We recently uncovered some molecular mechanisms by which fungus-induced activation of LRRK2 mediates experimental SAP. In this review, we summarize the role played by LRRK2 in the development of SAP.

## 2 Bacterial and fungal infection and SAP

Pancreatic necrosis is a hallmark of SAP (1–3, 9). Necrotic pancreatic tissues, which appear early in the clinical course of SAP, function as platforms for dissemination of bacteria and fungi leading to elevation in serum levels of lipopolysaccharide (LPS) and β-D glucan (3, 9, 10). Pancreatic necrosis is classified as acute necrotic collection (ANC) and walled-off necrosis (WON). ANC is a non-sequestrated and immature form of pancreatic necrosis generated within 4 weeks after the onset of acute pancreatitis (11, 12). ANC, which is usually visualized as a peripancreatic fluid collection on computed tomography, can progress to a mature and encapsulated form of pancreatic necrosis called WON (11, 12).

**Figure 1:**
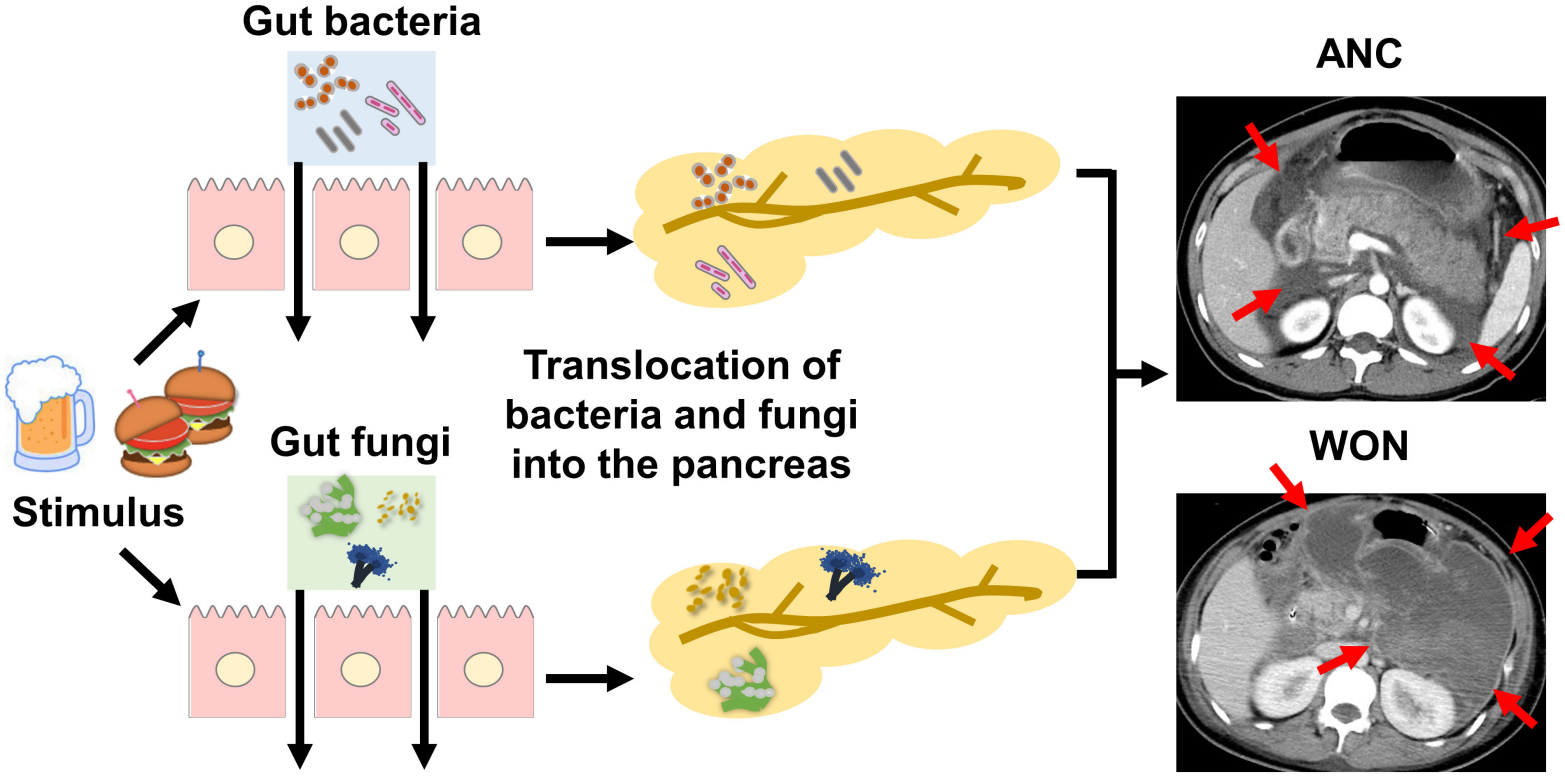
Association between pancreatic colonization of gut bacteria or fungi and severe acute pancreatitis. Alcohol consumption or high-fat diet triggers the development of acute pancreatitis. Gut bacteria or fungi migrate to the pancreas due to increased intestinal permeability associated with acute pancreatitis. Pancreatic colonization by gut bacteria or fungi promotes the development of SAP characterized by pancreatic necrosis. Pancreatic necrosis is classified as an acute necrotic collection (ANC, arrows) or walled-off necrosis (WON, arrows) as shown in contrast-enhanced dynamic computed tomography. ANC is an immature form of pancreatic necrosis, whereas WON is a mature encapsulated form of pancreatic necrosis. Pancreatic colonization by gut bacteria or fungi is involved in the maturation process from ANC to WON.

Pancreatitis is a unique form of inflammation in which the initial pancreatic injury leads to alterations in intestinal permeability, which enables gut microorganisms to enter the pancreas (1, 3, 13). The invasion of pancreatic tissue by gut bacteria, followed by dissemination of the organisms to distant organs is linked to the development of SAP, which is characterized by pancreatic necrosis and multiple organ failure (9, 10). Bacterial infection of pancreatic tissues is associated with the development of ANC or WON and progression of ANC to WON (1–3, 9) (**Figure 1**). Gram-negative rods derived from the gut is the most frequently isolated type of bacteria in necrotic pancreatic tissues and the peripheral blood of patients with SAP (14). Furthermore, colonization of gut bacteria in ANC and WON is a critical event leading to bacterial dissemination to the distant organs (15). Thus, accumulating clinical evidence strongly suggests that translocation of gut bacteria to the pancreas and subsequent colonization and infection is an essential step in the development of SAP with ANC or WON. This notion is supported by evidence from recent studies employing 16S ribosomal RNA (16S rRNA) sequencing conducted by Li et al. (16). They detected bacterial DNA in peripheral blood samples of approximately 70% of patients with acute pancreatitis, a higher percentage of patients with SAP (16). Moreover, the bacterial species isolated from the peripheral blood were gut-colonizing bacteria (16).

Similar to bacterial infection, fungal infection in ANC or WON is associated with SAP (3, 17–19). Pancreatic fungal infection is detected in approximately 40–50% of patients with WON using conventional culture methods, and patients with *Candida* infection display a higher mortality rate than those without *Candida* infection (17). Consistent with this finding, a retrospective study by Werge et al. (20) showed isolation of fungi from the pancreas of 57 of 123 patients (46.3%) with WON. Additionally, *Candida* species are frequently isolated in the peripheral blood of patients with SAP as assessed by culture methods or β-D glucan antigenemia (3, 17–19). Thus, the clinicopathological data suggest that pancreatic fungal infection is associated with the development of WON in a substantial proportion of patients with SAP. Given that *Candida* species are the predominant gut fungi (21), it is likely that migration of gut fungi to the pancreas due to an impaired intestinal barrier underlies the pathogenesis of SAP as is the case of bacterial colonization of the pancreas (**Figure 1**). One question that arises is whether ANC and WON are caused by solitary bacterial or fungal infection, or by bacterial and fungal co-infection. Fungal infection may be a relatively late event because many cases of fungal pancreatic infection are associated with preceding systemic administration of antibiotics (3). However, conventional culture methods were used in previous studies. Thus, the sequence of bacterial and fungal infection and the interplay between bacteria and fungi, both of which may occur in the pancreas with ANC or WON, are poorly understood. Therefore, sensitive next-generation sequencing studies targeting bacterial 16S rRNA and fungal ribosomal DNA internal transcribed spacer (ITS) are required.

## 3 Trypsinogen activation and the innate immune response to intestinal bacteria in acute pancreatitis

Intrapancreatic activation of trypsin followed by the autodigestion of the pancreatic tissues underlies the pathogenesis of acute pancreatitis (1, 22). Cholecystokinin secretion is induced by alcohol consumption or a high-fat diet (1, 22). Over-activation of cholecystokinin receptor (CCKR) by pancreatic acinar cells, induced by alcohol consumption or a high-fat diet, causes excessive production of pancreatic digestive enzymes, resulting in intrapancreatic transformation of inactive trypsinogen to trypsin (1, 22). This trypsin-centered autodigestion view is supported by studies of human hereditary pancreatitis (23). Gain-of-function mutations in *PRSS1*, which encodes trypsinogen and leads to overproduction of trypsin, have been identified as a cause of recurrent pancreatitis (23). In contrast, loss-of-function mutations in *SPINK1*, which encodes pancreatic secretary trypsin inhibitor, are responsible for hereditary recurrent pancreatitis (23). In addition, repeated injection of supramaximal doses of cerulein, a CCK agonist, causes acute pancreatic injury accompanied by elevated serum amylase and lipase levels (24). Thus, strong support for the trypsin-centered autodigestion view comes from both human pancreatitis and experimental animal studies. However, recent studies using mice deficient in the T7 isoform of trypsinogen, which corresponds to human cationic trypsinogen, provide evidence that mice experience experimental acute and chronic cerulein-induced pancreatitis even in the absence of T7 trypsinogen (25, 26). Thus, acute pancreatitis cannot be explained by intrapancreatic activation of trypsinogen alone. Recent studies have shed light on the innate immune responses caused by the migration of gut bacteria and fungi to the pancreas.

**Figure 2:**
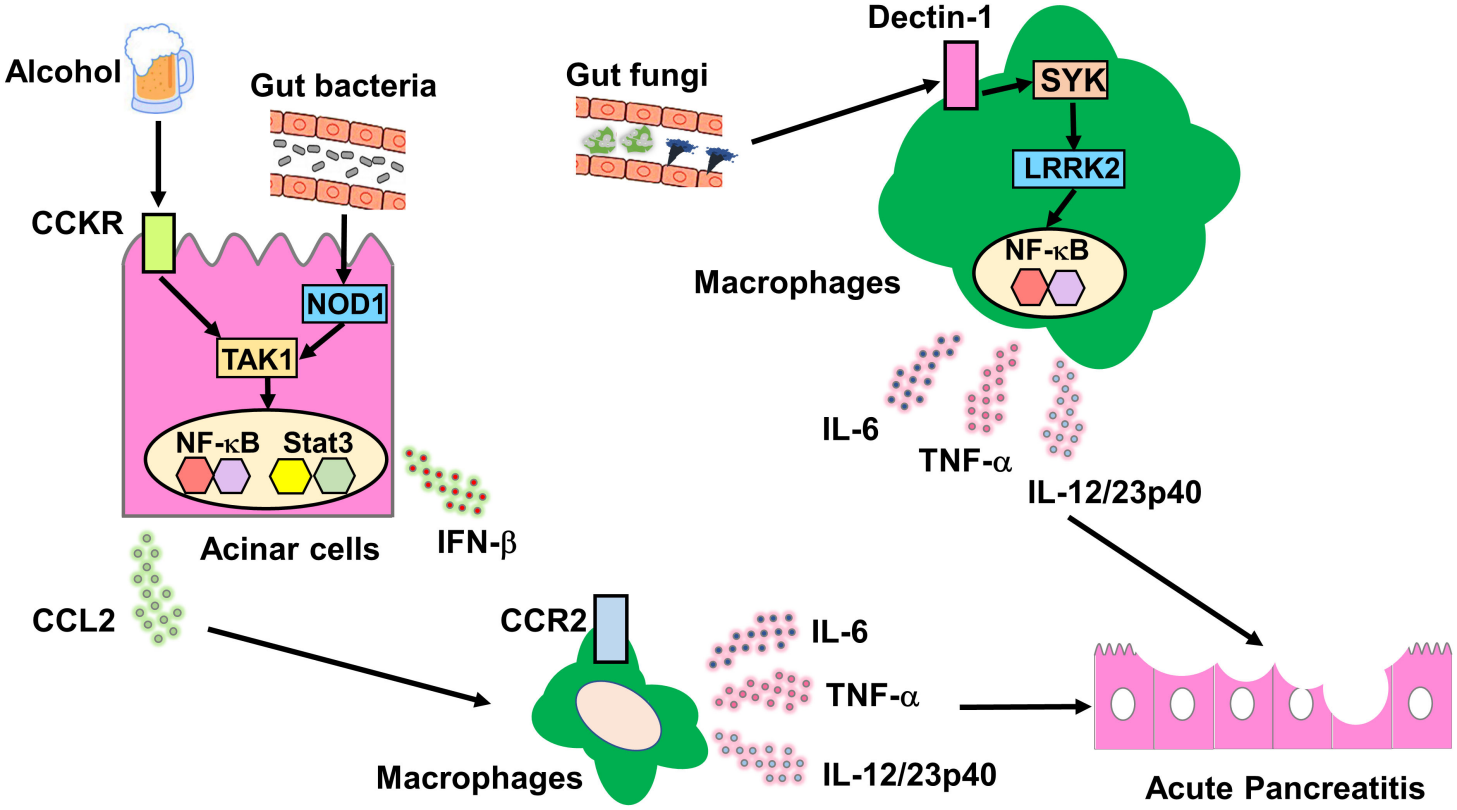
Molecular mechanisms accounting for the association between acute pancreatitis and innate immunity driven by gut bacteria or fungi. Molecular mechanisms underlying the association between acute pancreatitis and bacterial or fungi-driven innate immunity. Cholecystokinin receptor (CCKR) activation in pancreatic acinar cells is induced by excessive alcohol consumption or a high-fat diet. Gut bacteria that translocate to the pancreas are recognized by intracellular nucleotide-binding oligomerization domain 1 (NOD1) expressed in pancreatic acinar cells. Signaling pathways mediated by CCKR and NOD1 merge at the level of TGF-β-activated kinase 1 (TAK1) to induce synergistic activation of nuclear factor-κB (NF-κB). Co-stimulation with CCKR and NOD1 promotes the secretion of C-C motif chemokine ligand 2 (CCL2) by pancreatic acinar cells. CCL2 production by pancreatic acinar cells causes pancreatic migration of C-C chemokine receptor type 2 (CCR2)^+^ macrophages producing interleukin-6 (IL-6) and tumor necrosis factor alpha (TNF-α). Gut fungi that translocate to the pancreas are recognized by cell-surface Dectin-1 expressed in macrophages and dendritic cells. The Dectin-1-spleen tyrosine kinase (SYK)-leucine-rich repeat kinase 2 (LRRK2) axis induces production of proinflammatory cytokines including IL-6 and TNF-α through nuclear translocation of NF-κB subunits.

We evaluated the role played by gut bacteria-induced innate immunity in experimental acute pancreatitis (**Figure 2**). We found that mice deficient in NOD1 and TLR4 were resistant to cerulein-induced acute pancreatitis and that bowel sterilization by a broad range of antibiotics prevented the development of acute pancreatitis (4–6). Pancreatic acinar cells produce large amounts of proinflammatory cytokines, including interferon beta (IFN-β) and chemokines (C-C motif chemokine ligand 2, CCL2) on recognition of invading gut bacteria by intracellular NOD1. Importantly, CCKR and NOD1 signaling pathways act synergistically at the level of TGF-β-activated kinase 1 (TAK1), a shared downstream signaling molecule of both pathways, to promote nuclear translocation of nuclear factor-κB (NF-κB) subunits. Transactivation of NF-κB and signal transducer and activator of transcription 3 (Stat3) induced by signaling pathways mediated by CCKR, NOD1, and IFN-β leads to the production of a large amount of CCL2 by pancreatic acinar cells (4, 6). CCL2 in turn attracts C-C chemokine receptor type 2 (CCR2) macrophages producing IL-6 and TNF-α to the pancreas. Thus, the studies employing cerulein-induced acute pancreatitis confirm that cross-talks between pancreatic acinar cells and macrophages, leading to innate proinflammatory cytokine and chemokine responses, are associated acute pancreatic injury (1). Taken together, accumulating evidence from clinical and basic studies support the notion that innate immunity caused by recognition of gut bacteria by NOD1 plays an indispensable role in the development of SAP.

## 4 LRRK2 and proinflammatory cytokine responses

LRRK2 is a multidomain protein composed of armadillo, ankyrin repeat region, leucine-rich repeat, Ras of complex proteins (ROC), C-terminal ROC, mitogen-activated protein kinase, and WD40 protein-protein interaction domains (8). LRRK2 phosphorylates substrates through its kinase activity to regulate intracellular vesicle trafficking, cellular organelle homeostasis, and autophagy (8). Dysregulated function of LRRK2 causes human disease, as shown in genome-wide association studies in which polymorphisms in *Lrrk2* are associated with PD and CD (8). LRRK2 is expressed mainly in macrophages and DCs (7, 8). Macrophages and DCs are major cellular sources of proinflammatory cytokines that sense microbial components through PRRs, including TLRs, NOD-like receptors, and Dectin-1 (4, 27–29).

LRRK2 activation has been implicated in PRR-mediated proinflammatory cytokine responses. Macrophages from LRRK2-intact and LRRK2-deficient cells produce the same amount of proinflammatory cytokines on stimulation with a broad range of TLR ligands (30, 31). Takagawa et al. (7) examined PRR-mediated signaling pathways using bone marrow-derived DCs (BMDCs) from LRRK2-intact and LRRK2-transgenic (LRRK2-TG) mice, the latter of which overexpress LRRK2 in both hematopoietic and non-hematopoietic cells. They found that compared with LRRK2-intact BMDCs, BMDCs overexpressing LRRK2 produce higher amounts of TNF-α on stimulation with Dectin-1 ligands, but not on stimulation with TLR ligands. Mechanistically, LRRK2 mediates proinflammatory cytokine responses through interaction with TAK1 followed by nuclear translocation of NF-κB subunits (7). Such enhanced proinflammatory cytokine responses to Dectin-1 ligands predispose LRRK2-TG mice to experimental colitis and thus provide a mechanistic link between polymorphisms in LRRK2 and CD (7). Considering the pivotal role of Dectin-1 in fungal sensing, the study clearly shows that excessive proinflammatory cytokine responses triggered by Dectin-1-LRRK2 pathways underlie the immunopathogenesis of CD.

## 5 LRRK2 and acute pancreatitis

Dectin-1 functions as a PRR for fungal cell wall components including zymosan and β glucans (27, 32). These findings prompted us to examine whether the activation of LRRK2 by Dectin-1 on detecting fungal cell wall components mediates the development of SAP through proinflammatory cytokine responses (**Figure 2**) (33).

We used an acute pancreatitis model in which hourly intraperitoneal injections of cerulein was performed eight times over 2 consecutive days (33). Blockade of LRRK2-mediated signaling pathways by its specific inhibitor attenuated the development of acute pancreatitis and reduced pancreatic accumulation of T cells, macrophages, and DCs, which effects were accompanied by diminished pancreatic production of proinflammatory cytokines such as IL-6 and TNF-α. In contrast, compared with LRRK2-intact mice, LRRK2-TG mice exhibited a more severe form of acute pancreatitis characterized by elevated levels of amylase, pancreatic accumulation of immune cells, and destruction of the pancreatic acinar architecture. Importantly, SAP in LRRK2-TG mice was driven by NF-κB-mediated proinflammatory cytokine responses. LRRK2-TG mice display increased LRRK2 expression in macrophages, DCs, and pancreatic acinar cells. BM transplantation (BMT) experiments were conducted to determine whether LRRK2 expression in hematopoietic or non-hematopoietic cells mediates SAP in LRRK2-TG mice. The BMT studies clearly showed that LRRK2 of hematopoietic cell origin plays a critical role in the development of SAP. Taken together, these studies provide evidence that LRRK2 expressed in macrophages and DCs mediates SAP through proinflammatory cytokine responses.

We next addressed whether the proinflammatory cytokine responses causing SAP are driven by the translocation of gut bacteria and/or fungi to the pancreas in LRRK2-TG mice (33). LRRK2-TG mice were treated with a broad range of antibiotics and antifungal agents to deplete commensal bacteria and fungi, respectively. Depletion of the gut mycobiome, but not the bacterial flora, resulted in protection from SAP in LRRK2-TG mice. Protection from SAP achieved by the depletion of mycobiome was accompanied by decreased proinflammatory cytokine responses and immune cell infiltration in the pancreas. Pancreatic macrophages and DCs isolated from LRRK2-TG mice displaying SAP produced large amounts of IL-6 and TNF-α on stimulation with Dectin-1 ligands, but not TLR ligands, as compared with those from LRRK2-intact mice. Moreover, blockade of Dectin-1-mediated signaling pathways by administration of a spleen tyrosine kinase (SYK) inhibitor attenuated the development of SAP in LRRK2-TG mice (32). Collectively, these results strongly suggest that LRRK2 activation mediates experimental SAP through proinflammatory cytokine responses driven by sensing of fungi by Dectin-1.

These findings regarding the involvement of the Dectin-1-LRRK2 axis in the development of experimental SAP are reminiscent of pathogenic roles played by Dectin-1 and mannose-binding lectin (MBL) in pancreatic ductal adenocarcinoma (PDAC) (34–37). Landmark studies by Daley et al. (35) showed that intratumor accumulation of macrophages expressing Dectin-1 was present in human and murine PDAC tissue, and ligation of Dectin-1 resulted in a tolerogenic microimmune environment. Mycobiome composition studies targeting fungal ITS1 revealed greater amounts of fungal DNA in human and murine PDAC tissues than in normal pancreatic tissue (36). PDAC tissue are enriched with *Malassezia* species, which migrate from the gut lumen to the pancreas via the ampulla of Vater. Pancreatic colonization by *Malassezia* spp. promotes PDAC by the activation of complement cascades initiated by the recognition of fungal wall glycans by MBL (36). Alam et al. (34), reported that in PDAC, *Malassezia* spp. lead to the release of IL-33 through Dectin-1-mediated sensing of fungal cell wall components to generate tolerogenic microimmune environments. Thus, emerging evidence suggests that the mycobiome-Dectin-1-immune axis may be a suitable therapeutic target for treating PDAC. In keeping with the PDAC studies, our studies shed light on the pathogenic role played by the mycobiome-Dectin-1-LRRK2 axis in the development of SAP. However, it should be noted that pathogenic fungal species corresponding to the pathogenic role of *Malassezia* spp. in PDAC, have not been identified. The mycobiome analyses targeting ITS1 did not identify commensal fungi associated with the development of SAP in the stool of LRRK2-TG mice (33). Although no marked differences in commensal fungal populations in the stool were observed between LRRK2-intact and LRRK2-TG mice, a significant difference was observed between plant and mushroom-derived fungal species. At present, it is not known whether LRRK2 overexpression causes excessive proinflammatory cytokine responses leading to SAP on exposure to dietary fungi. However, exposure to dietary fungi might be involved in the development of SAP because dietary fungal exposure induces accumulation of cytotoxic T cells in CD (38).

TAK1 is a scaffold signaling molecule shared by signaling pathways mediated by CCKR and NOD1 (1, 4, 6). TAK1 activation is required to induce optimal activation of NF-κB (1, 4, 6). Synergistic interactions between signaling pathways mediated by CCKR and NOD1 underlie the pathogenesis of acute pancreatitis associated with bacterial translocation. Given that TAK1 is a downstream signaling molecule of LRRK2 (7), synergistic interaction between CCKR and Dectin-1/LRRK2 operating in pancreatic acinar cells might be involved in the development of SAP associated with fungal colonization. If this is the case, TAK1 may be a potential therapeutic target for treating SAP complicated by bacterial and/or fungal infection.

## 6 Conclusions

Translocation of fungi to the pancreas due to impaired intestinal barrier function is associated with the development of SAP. In experimental studies, the Dectin-1-LRRK2 axis is activated by recognition of fungi invading the pancreas, mediating the development of SAP through proinflammatory cytokine responses. The available evidence suggests that the fungi-Dectin-1-LRRK2 axis is a potential therapeutic target for SAP. However, human studies addressing the activation status of Dectin-1 and LRRK2 are required to confirm this hypothesis. *Lrrk2* mutations are linked to PD and CD, both of which are driven by an excessive immune response to gut microorganisms. Therefore, the association between *Lrrk2* polymorphisms and SAP also needs to be investigated in future studies. Although further human studies are required to confirm the linkage between the fungi-Dectin-1-LRRK2 axis and SAP, phase III clinical trials of LRRK2 inhibitors for treating PD are ongoing (39). Insights obtained from clinical trials on PD might open the way for clinical application of LRRK2 inhibitors in treating SAP.

## Author contributions

YO: Writing – original draft, Writing – review & editing. KM: Writing – original draft, Writing – review & editing. MK: Writing – original draft, Writing – review & editing. TW: Writing – original draft, Writing – review & editing.
